# Preparation and Properties of Fe-Based Double Perovskite Oxide as Cathode Material for Intermediate-Temperature Solid Oxide Fuel Cell

**DOI:** 10.3390/molecules29225299

**Published:** 2024-11-09

**Authors:** Liangmei Xue, Songbo Li, Shengli An, Ning Li, Huipu Ma, Mengxin Li

**Affiliations:** 1 School of Chemistry and Chemical Engineering, Inner Mongolia University of Science and Technology, Baotou 014010, China; 15941277965@163.com (L.X.); ln19981213@outlook.com (N.L.); g18347714572@hotmail.com (H.M.); lmx3210@hotmail.com (M.L.); 2 School of Materials and Metallurgy, Inner Mongolia University of Science and Technology, Baotou 014010, China; san@imust.edu.cn

**Keywords:** IT-SOFC, Fe-based, Mo doping, electrochemical performance

## Abstract

Double perovskite oxides with mixed ionic and electronic conductors (MIECs) have been widely investigated as cathode materials for solid oxide fuel cells (SOFCs). Classical Fe-based double perovskites, due to their inherent low electronic and oxygen ionic conductivity, usually exhibit poor electrocatalytic activity. The existence of various valence states of B-site ions modifies the material’s catalytic activity, indicating the possibility of the partial substitution of Fe by higher-valence ions. LaBaFe_2−x_Mo_x_O_5+δ_ (*x* = 0, 0.03, 0.05, 0.07, 0.1, LBFM_x_) is used as intermediate-temperature solid oxide fuel cell (IT-SOFC) cathode materials. At a doping concentration above 0.1, the Mo substitution enhanced the cell volume, and the lattice expansion caused the formation of the impurity phase, BaMoO_4_. Compared with the parent material, Mo doping can regulate the oxygen vacancy concentration and accelerate the oxygen reduction reaction process to improve the electrochemical performance, as well as having a suitable coefficient of thermal expansion and excellent electrode stability. LaBaFe_1.9_Mo_0.1_O_5+δ_ is a promising cathode material for IT-SOFC, which shows an excellent electrochemical performance, with this being demonstrated by having the lowest polarization resistance value of 0.017 Ω·cm^2^ at 800 °C, and the peak power density (PPD) of anode-supported single-cell LBFM_0.1_|CGO|NiO+CGO reaching 599 mW·cm^−2^.

## 1. Introduction

The solid oxide fuel cell (SOFC) is an electrochemical device that directly converts chemical energy in fuel into electrical energy through an electrode reaction and is a very promising new energy technology for efficient power generation based on existing energy supply systems [1]. Considering the high operating temperature of traditional SOFCs, cell encapsulation is hard to perform, and the production cost is high. Therefore, the development of IT-SOFCs that can operate at medium temperature is one of the current research hotspots [2]. However, as the operating temperature decreases, the polarization impedance of the cathode increases significantly, thus mainly restricting the electrochemical performance of IT-SOFCs [3]. Therefore, researchers have focused on improving the output performance of SOFCs under medium-temperature conditions by studying excellent cathodes. Currently, for anode-supported SOFCs, the slow oxygen reduction reaction (ORR) at the cathode dominates the polarization loss of the cell [4,5]. The lack of efficient, low-cost, and stable cathode materials is an urgent problem that needs to be solved. In recent years, the LnBaCoO_5+δ_-type double perovskite oxide, which contains the rare earth element Ln, has been widely studied as an SOFC cathode material because of its special layered structure, resulting in its excellent conductivity and oxygen transport properties [6]. However, Co-based double perovskites generally encounter problems such as a high average thermal expansion coefficient (TEC), poor stability, and high cost [7,8]. The Fe-based perovskite oxide is among the most potential alternative materials for cobalt-based cathodes because of its high reserves in nature, low cost, and good resistance to CO_2_ and vapor [9]. However, it suffers from poor electrocatalytic activity, thus requiring the optimization of more materials as IT-SOFC cathode materials.

Considering the existence of various valences of B-site transition metal ions, the valence of ions can be adjusted intuitively by B-site doping to change the crystal structure, affect the oxygen ion diffusion, and improve the catalytic activity. In addition, the B-site doping of the perovskite structure oxide with stable ions can improve the stability of the cathode material in a high-temperature environment and cathode atmosphere (CO_2_ and humid air) and effectively reduce the TEC of the cathode material. The most common doping materials are Cu, Fe, Ni, and Mn [10,11]. The incorporation of high-valence cations such as Nb^5+^, Ta^5+^, and W^6+^ can effectively stabilize the phase structure of peroxides, reduce the TEC, improve the thermal matching with other devices, enhance the catalytic activity, increase the output power, and raise the life of cells [12,13,14]. Some Fe- and Co-based peroxides are often doped with Mo to improve the electrochemical performance [15,16]. Therefore, in this paper, LBFM_x_ (*x* = 0, 0.03, 0.05, 0.07, 0.1) was prepared by doping high-valence Mo at the B position, and the effects of Mo doping on the phase structure, thermal expansion characteristics, conductivity, and electrochemical properties were studied in detail.

## 2. Results and Discussion

**Figure 1:**
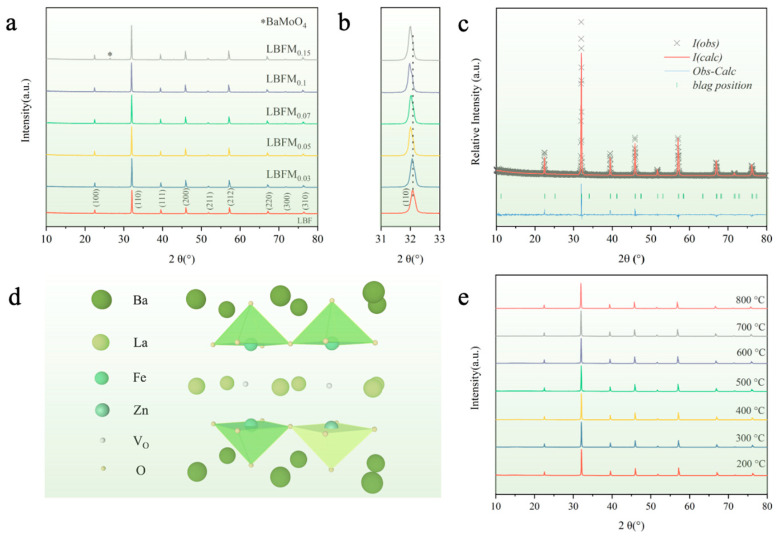
(**a**) XRD patterns of the cathode material LBFM_x_; * corresponds to peak of BaMoO_4_; (**b**) localized magnification; (**c**) Rietveld refinement pattern of the LBFM_0.1_ sample; (**d**) crystal structure of LBFM_0.1_; and (**e**) HT-XRD patterns of the LBFM_0.1_.

Figure 1a shows the XRD patterns of the sample LBFM_x_ (*x* = 0, 0.03, 0.05, 0.07, 0.1, 0.15) after calcination at 1100 °C. No impurity phase was obtained when the doping amount is less than 0.1, indicating that LBFM_x_ is in a pure double perovskite phase [17]. The XRD diffraction peaks of LBFMx gradually shifted to a small angle with the increase of Mo doping as shown in Figure 1b, which indicates that the cell volume of LBFMx gradually increases and the lattice expands with the increase of doping. The impurity phase BaMoO_4_ (PDF.#8-455) was detected as the doping amount was increased to over 0.1 [18]. The crystal structure of LBFM_x_ was determined by refining the XRD patterns by using the GSAS/EXPGUI software (PC-GSAS) (Figure 1c and Figure S1), which showed a good match with the XRD pattern, and the refinement results are listed in Table S1. The results show that the cell parameter of LBFM_x_ gradually increased with the increase in doping amount, and the cell volume increased, because the Mo^6+^ radius (0.59 Å) and Fe^4+^ radius (0.585 radius) are close [19]. The Fe^3+^ content increased after the B-site Fe was replaced by Mo, thus increasing the cell volume. The results show that LBFM_x_ samples have the same structure as the undoped LBF, indicating that Mo doping does not change the original crystal structure. Figure 1d shows the crystal structure of LBFM_0.1_. The LnO and BaO layers are arranged in alternating layers of -BaO-FeMoO_2_-LaO_δ_-FeMoO_2_-BaO- along the C-axis, and oxygen vacancies mainly form and migrate in the LaO_δ_ layer. The HT-XRD patterns of the material under an air atmosphere in the range of 200 to 800 °C were tested to assess the thermal stability of the materials, and Figure 1e shows that the materials are all in a single phase, indicating that the materials have a good thermal stability.

**Figure 2:**
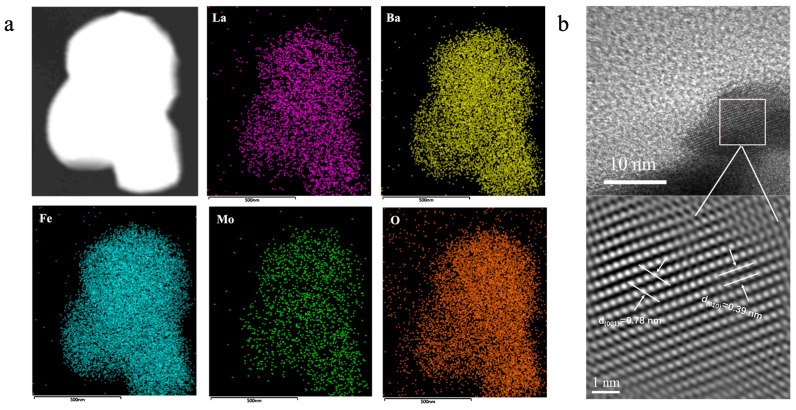
(**a**) TEM image and EDS maps of the LBFM_0.1_ powder; and (**b**) HR-TEM image of LBFM_0.1_.

Figure 2a shows the energy-dispersive spectroscopy (EDS) diagram of LBFM_0.1_ cathode powder attached to the CGO electrolyte, showing that La, Ba, Fe, Mo, and O are evenly distributed, and no element aggregation is found. This finding confirms that Mo successfully replaces Fe at the B-site. LBFM_x_ has a tetragonal encapsulated crystal structure in the P4/mmm space group, and sample LBFM_0.1_ was characterized using transmission electron microscope as shown in Figure 2b, where the lattice spacings of 0.39 nm and 0.78 nm correspond to the (010) and (001) crystallographic planes, respectively.

During the operation of SOFCs, oxygen ions are transferred from the cathode layer to the electrolyte layer; consequently, the poor chemical compatibility between the cathode layer and the electrolyte may create an insulating layer at the cathode/electrolyte interface, thus greatly reducing the performance of SOFCs [20]. The LBFM_0.1_ sample and CGO powder were mixed at a mass ratio of 1:1 and sintered at 1100 °C for 5 h. The sintered CGO-LBFM0.1 mixture was studied by XRD, and the results are shown in Figure S2. Compared with the XRD data of the single-phase LBFM_0.1_ and CGO, the diffraction peak of the mixture after sintering has no obvious shift, and no new phase formed after high-temperature heating, indicating that it has a good chemical compatibility with the electrolyte CGO at high temperature.

**Figure 3:**
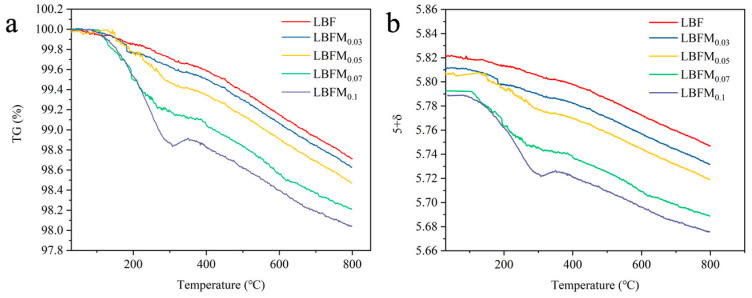
(**a**) TG curve of LBFM_x_; and (**b**) curve of oxygen content (5 + δ) in LBFM_x_ at the air atmosphere.

The TGA curves of the LBFM_x_ (*x* = 0, 0.03, 0.05, 0.07, 0.1) series cathode powders from 30 °C to 800 °C are shown in Figure 3a. The decrease in mass between 30 °C and 300 °C is attributed to the loss of water and gas at the surface of the cathode material [21]. At temperatures higher than 300 °C, this phenomenon is mainly caused by the escape in lattice oxygen accompanied by the generation of oxygen vacancies [22], resulting in a decrease in mass, as shown in Equation (1).

(1) $${{2\mathrm{Fe}}^{\cdot }}_{\mathrm{Fe}}{+O}_{O}^{\times }\Leftrightarrow {2\mathrm{Fe}}_{\mathrm{Fe}}^{\times }{+V}_{O}^{¨}+\frac{1}{2}O_{2}↑$$

${{\mathrm{Fe}}^{\cdot }}_{\mathrm{Fe}}$, ${\mathrm{Fe}}_{\mathrm{Fe}}^{\times }$, $O_{O}^{\times }$, and $V_{O}^{¨}$ represent Fe^4+^, Fe^3+^, and the lattice oxygen and oxygen vacancy. As shown in Figure 3a, the increase in doping amount will increase the weight loss because of the water absorbed by the material and oxygen adsorbed on the surface, and the increase in surface oxygen vacancy will decrease the weight. According to the TGA curve from 30 °C to 800 °C, the weight loss rate of a series of cathode materials is only between 1.3% and 2.0% during heating, confirming that the prepared materials have a high structural stability. The iodimetric titration results show that the oxygen non-stoichiometric ratio of the sample decreased with the increase in doping amount (Table S2). The change curve of the oxygen content and temperature of LBFM_x_ was obtained by combining the TGA data, as shown in Figure 3b. The oxygen content gradually decreased with the increase in doping amount, indicating that the oxygen vacancy of the material gradually increased after substitution. Initially, the oxygen content of the sample remains unchanged with the increase in temperature, possibly because oxygen ions are frozen in the lattice at low temperature [23]. With the further increase in temperature, the oxygen content of the sample gradually decreases, and this phenomenon is related to the release of lattice oxygen caused by high temperature.

**Figure 4:**
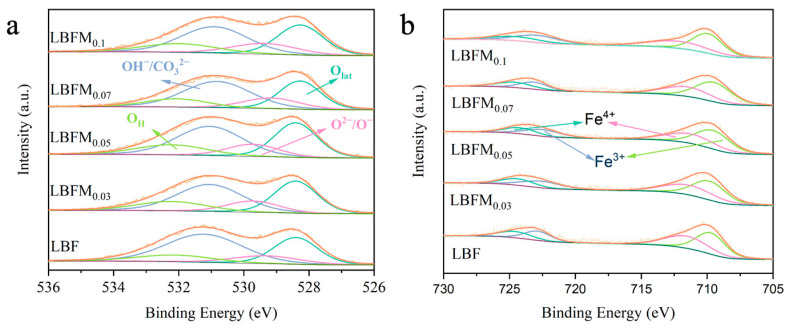
XPS curve of LBFM_x_; (**a**) O1s, and (**b**) Fe2p.

The chemical valence states of surface ions of LBFM_x_ (*x* = 0, 0.03, 0.05, 0.07, 0.1) series materials were analyzed by XPS, because the cationic oxidation state is an important factor that affects the catalytic activity of the cathode. As shown in Figure 4a, the high-resolution map of O1s can fit four different characteristic peaks, indicating the existence of different types of oxygen species [24]. The diffraction peak at 528.35 eV (±0.2 eV) can be attributed to lattice oxygen (*O_lat_*). The peak at 529.3–531.12 eV (±0.2 eV) is related to the adsorbed oxygen (O^2−^O^−^ and OH^−^/CO_3_^2−^), while the peak at 532.11 eV (± 0.2 eV) belongs to *O_vacancy_* and moisture oxygen (*O_H_*). The ratio between the adsorbed oxygen (*O_C_*) and lattice oxygen (*O_lat_*) reflects the content of surface oxygen vacancies. Therefore, the calculated *O_C_/O_lat_* ratio is listed in Table S3, and its ratio is relatively increased with the increase in doping amount, indicating that Mo can replace Fe at the B-site to obtain more oxygen vacancies and oxygen adsorption/dissociation active centers. In the LBF lattice, the holes generated by Fe^3+/^Fe^4+^ are electron carriers and provide Fe^3+^–O^2−^– Fe^4+^ electron transport paths for hole transport [25]. Therefore, the valence state of Fe in LBFM_x_ materials needs to be studied. Figure 4b shows the high-resolution XPS map and fitting curve of Fe2p at room temperature. The binding energy is decomposed into two different peaks at 710.2 eV (±0.15 eV) and 711.2 eV (±0.26 eV), corresponding to Fe^3+^2p_3/2_ and Fe^4+^2p_3/2_, respectively. The binding energies of 722.9 eV (±0.14 eV) and 724.2 eV (±0.25 eV) correspond to Fe^3+^2p_1/2_ and Fe^4+^2p_1/2_, respectively [26,27]. The percentage values of Fe^3+^ and Fe^4+^ are listed in Table S3. With the increase in the Mo doping amount, the content of Fe^4+^ decreased, and the average valence state of Fe gradually decreased.

**Figure 5:**
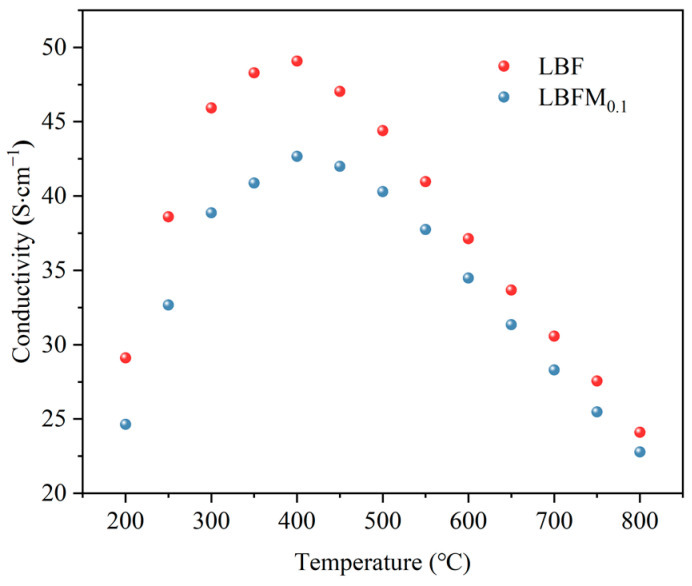
Conductivity of LBFM_x_ at air atmosphere.

For the understanding of the effect of Mo doping on oxygen ion and hole conduction, the conductivity of LBFM_x_ (*x* = 0 and 0.01) was measured from 200 °C to 800 °C in air, and the results are shown in Figure 5. At 400 °C, both samples showed an increasing trend of electrical conductivity with increasing temperature, which follows the small polaron conduction mechanism [28]. The electrical conductivity decreased with increasing temperature at 400–800 °C, because the oxygen content decreased with increasing temperature, and a large amount of lattice oxygen is released. Subsequently, the carrier concentration gradually decreased. At 400 °C, the conductivities of LBF and LBFM_0.1_ are 49 and 42 s·cm^−1^, respectively. The conductivity decreased with the increase in Mo doping concentration. This finding can be explained by the following reasons: According to the principle of charge conservation, Mo doping at the B-site causes a partial reduction of Fe^4+^ to Fe^3+^, which is confirmed by the XPS results, and this phenomenon leads to a decrease in the concentration of electron holes. Therefore, the conductivity of electrons decreased. Second, the electron conduction in peroxides follows the small-pole jump conduction mechanism and is conducted by the B^n+1^-O^2−^-B^n−1^ net structure, and the non-conductive Mo-O bond increases with the increase in Mo substitution, thus hindering the electron transport and decreasing the conductivity [19,29].

**Figure 6:**
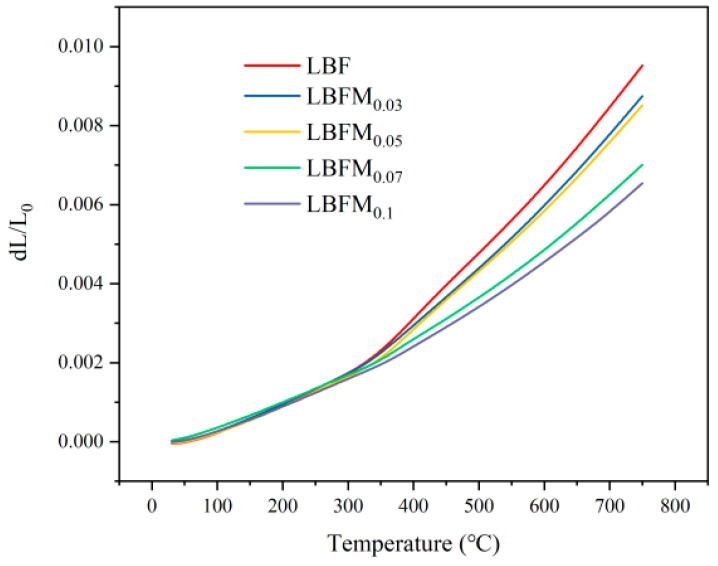
Thermal expansion curve of LBFM_x_ at air atmosphere.

Figure 6 shows the thermal expansion curves of LBFM_x_ at 30–750 °C in air. Based on the figure, the relationship curve between the thermal expansion degree (dL/*L*_0_) and temperature is not completely linear. The mechanism of the thermal expansion behavior of oxides is different at high and low temperature, and the thermal expansion at low temperature is related to physical expansion. However, the thermal expansion at the high-temperature range is influenced by both physical and chemical expansion. In addition, the chemical expansion is related to the reduction of Fe in the B-site and the formation of oxygen vacancies in the sample [18,30]. According to the figure, the thermal expansion of the sample increases with the increase in temperature, and the increase in TEC corresponds to the crystal expansion caused by harmonic atomic vibration. When the temperature is above 300 °C, the rapid increase in TEC is caused by the Fe decrease and the formation of oxygen vacancies in the sample. This process can be represented by Equation (1). As shown in Table S4, the average TEC of the sample gradually decreased with increasing Mo doping concentration, indicating that Mo doping increased the thermal stability. This increase in thermal stability can be attributed to the stronger binding strength of Mo–O (359.92 kJ·mol^−1^) than that of Fe–O (200.15 kJ·mol^−1^) [18]. Furthermore, the incorporation of Mo reduced the amount of Fe^4+^ in the sample, which also favored the TEC reduction. The average TEC values for LBFM_x_ (*x* = 0, 0.03, 0.05, 0.07, 0.1) at 30–750 °C are 13.2 × 10^−6^·K^−1^, 12.1 × 10^−6^·K^−1^, 11.8 × 10^−6^·K^−1^, 9.73 × 10^−6^·K^−1^, and 9.09 × 10^−6^·K^−1^. These values are compatible with the widely used CGO electrolyte materials (TEC = 11.9 × 10^−6^ K^−1^) and are substantially lower than those of cobalt-based peroxide materials, such as BaBi_0.05_Co_0.8_Nb_0.15_O_3-δ_ (19.6 × 10^−6^ K^−1^, 30–800 °C) and BaCo_0.7_Fe_0.2_Nb_0.1_O_3-δ_ (24.3 × 10^−6^ K^−1^, 50–800 °C) [31,32]. The incorporation of Mo in LaBaFe_2_O_5+δ_ significantly decreased the TEC, especially at the high–temperature range. The average TEC between 300 °C and 750 °C decreases from 21.2 × 10^−6^ K^−1^ to 14.5 × 10^−6^ K^−1^, corresponding to a change of 26%. It could improve the thermal compatibility between the cathode and electrolyte and improve the long-term cyclic stability of SOFCs.

**Figure 7:**
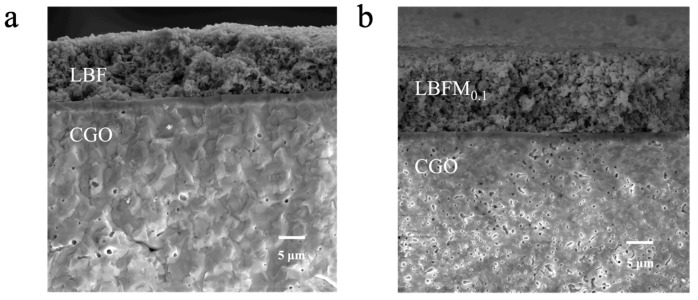
SEM of LBFM_x_|CGO section: (**a**) *x* = 0, and (**b**) *x* = 0.1.

Figure 7 and Figure S3 show the SEM images of symmetric cell sections LBFM_x_|CGO (*x* = 0 and 0.1). The samples are all loose and porous, which is conducive to gas diffusion, oxygen ion transport, and charge transfer. In addition, the cathode material is well-attached to the electrolyte CGO, and no obvious cracking and delamination was observed between the electrolyte and the cathode material, confirming that it has a good chemical compatibility with the electrolyte.

**Figure 8:**
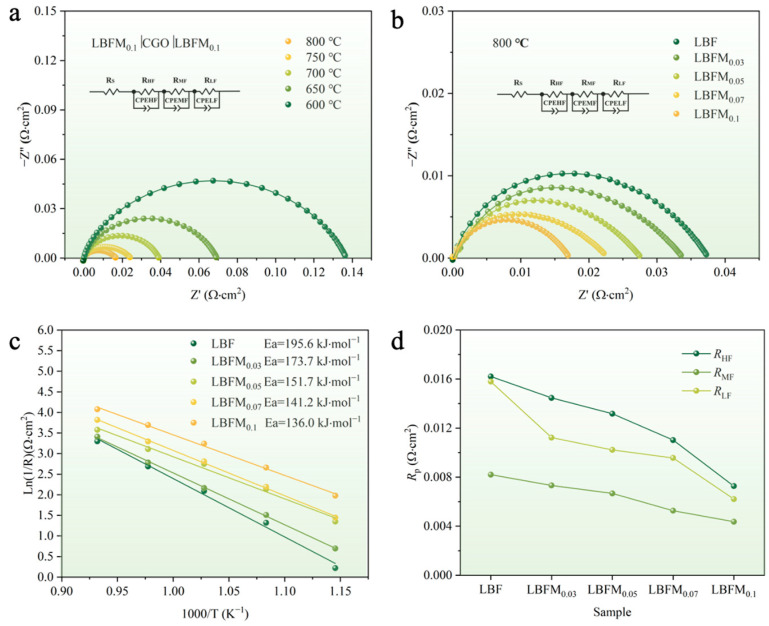
**(a**) Nyquist curve of LBFM_0.1_|CGO|LBFM_0.1_ in the range of 600–800 °C; (**b**) Nyquist curve of LBFM_x_|CGO|LBFM_x_ at 800 °C; (**c**) *R*_p_ Arrhenius diagram of LBFM_x_ in the range of 600–800 °C; and (**d**) *R_p_* of LBFM_x_ at 800 °C.

The activity and effectiveness of LBFM_0.1_ as an SOFC oxygen electrode were evaluated by preparing a symmetric cell with CGO as the electrolyte, and the sample was named LBFM_0.1_|CGO|LBFM_0.1_. The electrochemical impedance spectroscopy (EIS) curve measured in the range of 600–800 °C under the air condition is shown in Figure 8a. The equivalent circuit diagram *R_s_* (*R_HF_*//CPEHF *R_MF_*//CPEMF *R_LF_*//CPELF) was used to fit the measured AC impedance profile. *R_s_* represents the Ohmic resistance. *R*_HF_, *R*_MF_, *R*_LF_, and *R_p_* correspond to high-, intermediate-, and low-frequency polarization resistance and total resistance *R_p_* (*R_p_ = R_HF_ + R_MF_ + R_LF_*), respectively [33,34]. The effect of Mo doping on the electrochemical properties is obtained by preparing a symmetric cell LBFM_x_|CGO|LBFM_x_ (*x* = 0, 0.03, 0.05, 0.07, 0.1). The AC impedance spectra in the range of 600–800 °C were obtained. The above equivalent circuit diagram was used for fitting, and the results are shown in Table S5. Figure 8b shows the AC impedance spectrum at 800 °C. The *R_p_* value gradually decreased with the increase in doping amount, because Mo doping increases the oxygen vacancy concentration of the sample, promotes oxygen adsorption and deionization, improves surface oxygen exchange and oxygen ion diffusion, and is conducive to the ORR process [35,36]. Figure 8c shows the Arrhenius diagram of *R_p_* values of LBFM_x_ at 600–800 °C, with *E_a_* values of 195.6, 173.7, 151.7, 141.2, and 136 kJ·mol^−1^. This finding indicates that Mo substitution for Fe can promote the oxygen reduction reaction. The electrochemical reduction reaction of oxygen on the cathode surface and at the cathode/electrolyte interface are complex processes [37,38]. The specific role of Mo doping at the B-site was determined by calculating the *R_p_* values of LBFM_x_|CGO|LBFM_x_ at 800 °C in the high-, middle-, and low-frequency bands as shown in Figure 8d. Compared with LBF, the *R_p_* values of LBFM_0.1_ corresponding to the high-, middle-, and low-frequency region significantly decreased and is in the order of *R*_HF_ > *R_L_*_F_ > *R_M_*_F_, indicating that the transfer of oxygen ions is the rate control step of the ORR process.

**Figure 9:**
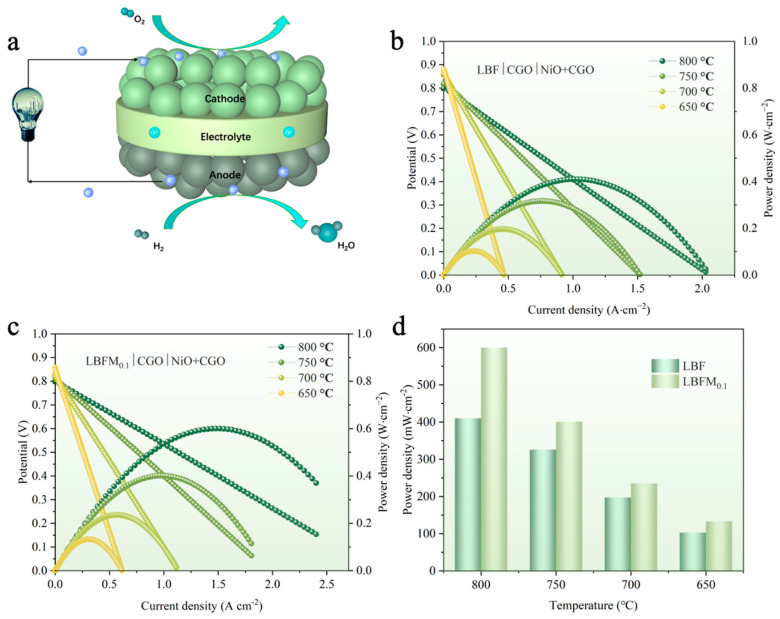
(**a**) Structure of the single cell; (**b**) I-V-P curve of the single cell LBF|CGO|NiO + CGO; (**c**) I-V-P curve of the single cell LBFM_0.1_|CGO|NiO + CGO; and (**d**) the PPD values of the single cell at different temperature.

The effect of doping Mo on the output performance of a single cell was compared by preparing the anodic supported single cell with CGO as the electrolyte, and the structure is shown in Figure 9a. By using humidified hydrogen as the fuel gas and having the cathode side in direct contact with air, a single-cell LBFM_x_|CGO|NiO + CGO (*x* = 0 and 0.1) was tested. The I–V–P curves at 650–800 °C are shown in Figure 9b,c. Figure 9d shows the measured peak power density (PPD) of a single cell at different temperatures. The PPD value of LBFM_0.1_|CGO|NiO + CGO is 599 mW·cm^−2^ at 800 °C, which is about 46% higher than that of LBF at the same temperature, possibly because of the low polarization resistance of LBFM_0.1_, indicating that the Mo substitution of Fe at the B-site improves the electrochemical performance [19]. Figure S4 shows the SEM image of the single-cell LBFM_0.1_|CGO|NiO + CGO after the test. No obvious delamination was observed between the components, indicating the good chemical compatibility and thermal matching between components. The electrolyte layer after sintering at high temperature is relatively dense, ensuring a high open-circuit voltage.

## 3. Experiment

### 3.1. Preparation

LBFM_x_ (*x* = 0, 0.03, 0.05, 0.07, 0.1) powder was prepared using the sol–gel method. La(NO_3_)_3_·6H_2_O (A.R.), Ba(NO_3_)_2_ (A.R.), Fe(NO_3_)_2_·9H_2_O (A.R.), and H_24_Mo_7_N_6_O_24_·4H_2_O (A.R.) were mixed according to the stoichiometric ratio, and then the appropriate amount of deionized water was added to the beaker and dissolved by stirring (all reagents are from Aladdin). Citric acid and EDTA were added as complexing agents, and the pH value of the solution was adjusted to 8 by adding an appropriate amount of ammonia. Finally, the solution was heated in a water bath at 80 °C and stirred continuously until the gel was formed. The gel was heated until spontaneous combustion to form a puffier black precursor, and the target powder was obtained after heating the precursor at 1100 °C for 5 h. Gd_0.1_Ce_0.9_O_1.95_ (CGO) powder was synthesized using the same preparation method, and the resulting precursor was heat-treated at 800 °C for 3 h to obtain a fluggy CGO electrolyte powder. The synthesized powder LBFM_x_ was pressed into cuboid-like strip samples at a pressure of 300 MPa and then sintered at 1200 °C for 5 h with dimensions of 29.5 mm × 6.0 mm × 0.5 mm.

### 3.2. Single-Cell Fabrication

The CGO powder was placed in a circular mold with a diameter of 12 mm and pressed into a circular sheet under a pressure of 200 MPa. After heating at 1450 °C for 5 h, a dense electrolyte sheet was obtained. The prepared cathode slurry was evenly coated on both sides of the electrolyte CGO by screen printing, dried, and finally kept at 1100 °C for 5 h. The symmetric battery LBFM_x_|CGO|LBFM_x_ (*x* = 0, 0.03, 0.05, 0.07, 0.1) for electrochemical impedance test was equipped with silver wire and silver paste for current collection. The anode-supported single cell was prepared using the common pressure method. The specific preparation process is as follows: the anode material (NiO-CGO) and the fluppy electrolyte powder were evenly spread into a circular mold with a diameter of 15 mm, and the two samples were dry-pressed to form at a pressure of 200 MPa. The pressed raw half battery was kept at 1450 °C for 5 h to obtain the anode-supported half battery. The cathode material was evenly coated on the half-cell (electrolyte CGO side) by screen printing. After drying, the single cell was obtained by sintering it in muffle furnace at 1100 °C for 5 h.

### 3.3. Characterization

The phase composition and crystal structure of the samples were analyzed using an X-ray diffractometer (XRD, Malvern Panalytical, Empyrean), and XRD equipped with Cu Kα radiation (40 kV, 40 mA, and λ = 1.5418 Å) at a scan rate of 5°/min and at the scan range of 10°–80°. The results were Rietveld-refined using the GSAS/EXPGUI software (PC-GSAS). The elemental distribution and lattice spacing of the material were also characterized using transmission electron microscopy (TEM, JEOL, 2100F, Tokyo, Japan). The oxygen content of the samples at different temperatures was characterized by iodine titration and thermogravimetry analysis (TGA, Netzsch, STA2500, Selb, Germany). The surface elemental valence states of the LBFM_x_ powder were analyzed by X-ray photoelectron spectroscopy (XPS, Thermo Scientific, EscaLab250Xi, Waltham, MA, USA). The linear expansion rate of the sample was measured using a thermal expander (Netzsch, DIL402C), and the average TEC of the sample was calculated. This test was carried out under air atmosphere at the test temperature range of 30–750 °C, and the heating rate was 5 °C·min^−1^. A scanning electron microscope (SEM, TESCAN, GAIA3, Brno, Czechia) was used to observe and analyze the micromorphology of the cross-section of the cell. An electrochemical workstation (Autolab, PGSTAT302N, Chișinău, Moldova) was used to test the conductivity of the material at 200–800 °C, and the electrochemical impedance spectrum of the symmetrical cell was obtained. The test frequency range was 0.1 Hz to 100 kHz, and the amplitude of the AC signal was 10 mV. In addition, the output power of a single cell was tested at 650–800 °C with an interval of 50 °C, where the cathode side was in direct contact with air and the anode side was continuously energized with humidified H_2_ (H_2_ + 3% H_2_O).

## 4. Conclusions

The present work focused on synthesizing and characterizing a Fe-based double perovskite oxide; the Fe at the B-site replaced by Mo does not change the original crystal structure; and LBFM_x_ all show a tetragonal double perovskite structure. With the increase in Mo doping amount, the cell parameters gradually increased, and the lattice was expanded. With the increase in Mo doping amount, the oxygen content gradually decreased, the oxygen vacancy concentration increased, and the content of Fe^4+^ decreased, resulting in the decrease in conductivity and carrier concentration. Given that Mo-O has a stronger binding strength than Fe-O, Mo doping increases the thermal stability of the sample, and the average TEC of the sample gradually decreases, indicating that Mo doping improves the thermal matching with the electrolyte CGO. Based on the increase in the oxygen vacancy concentration, the ORR process is improved, and the LBFM_x_ electrode shows excellent electrochemical performance. The *R_p_* values of the symmetric cell prepared with LBF and LBFM_0.1_ as cathodes are 0.04 and 0.017 Ω·cm^2^ at 800 °C, respectively, and the *R_p_* value of the optimized sample decreased by about 56.0%. The results show that Mo doping accelerates the transfer of oxygen ions. The PPD value of the anode supported single-cell LBFM_0.1_|CGO|NiO + CGO at 800 °C is 599 mW·cm^−2^. The above results indicate that LaBaFe_1.9_Mo_0.1_O_5+δ_ is a promising IT-SOFC cathode material.

## Data Availability

The data presented in this study are available on request from the corresponding author.

## Supplementary Materials

The following supporting information can be downloaded at: https://www.mdpi.com/article/10.3390/molecules29225299/s1, Figure S1: Rietveld refinement patterns of LBFMx samples; Figure S2: Rietveld refinement patterns of LBFMx samples; Figure S3: SEM of LBFMx|CGO section; Figure S4: SEM of the single cell section LBFM0.1|CGO|NiO+CGO; Table S1: Rietveld refinement results of LBFMx; Table S2: Oxygen content of LBFMx, oxygen vacancy, average valence of Fen+; Table S3: XPS percentage values for Fe and O elements; Table S4: Average coefficient of thermal expansion of LBFMx (K-1); Table S5: Polarization resistance of LaBaFe2-xMoxO5+δ (Ω·cm^2^).
