# Supplementary material for: Preparation and Properties of Fe-Based Double Perovskite Oxide as Cathode Material for Intermediate-Temperature Solid Oxide Fuel Cell
Source: Molecules. 2024 Nov 9;29(22):5299. doi: 10.3390/molecules29225299 (PMC11596265; doi:10.3390/molecules29225299)
Supplement: Supplementary file 1 [file molecules-29-05299-s001.zip › molecules-3246046-supplementary.pdf]

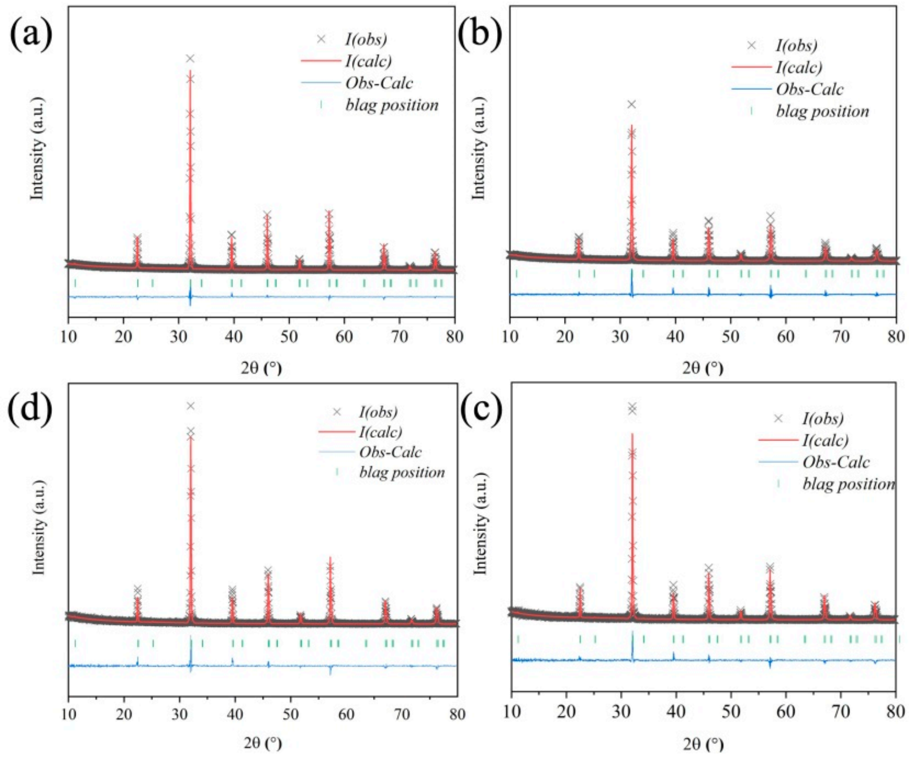

Figure S1. (a~d) Rietveld refinement patterns of $LBFM_x$ samples (x=0, 0.03, 0.05 and 0.07)

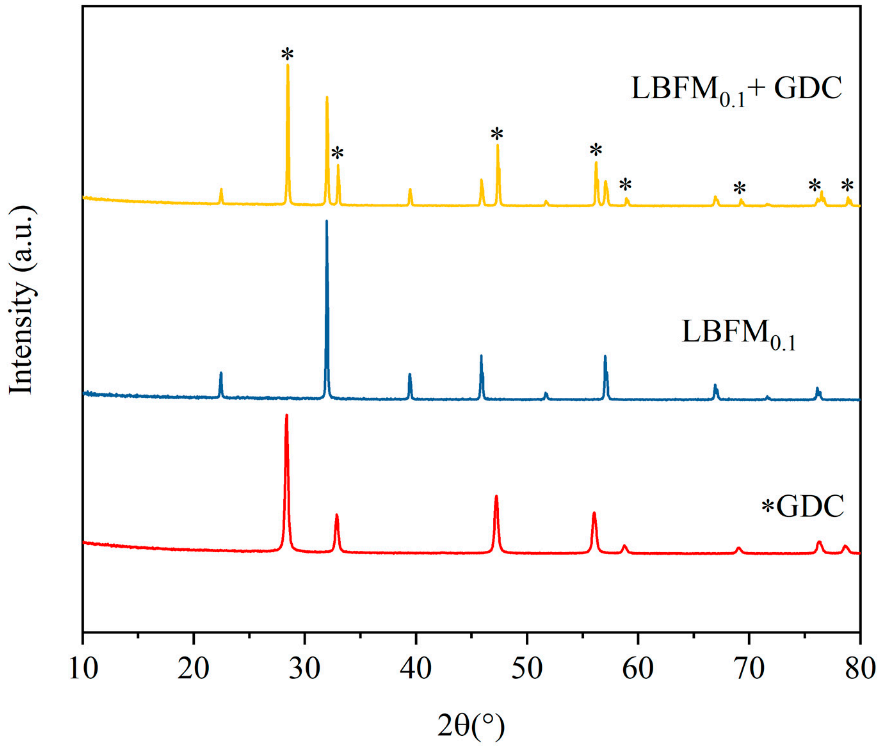

Figure S2. XRD patterns of the mixture $LBFM_{0.1}$|CGO

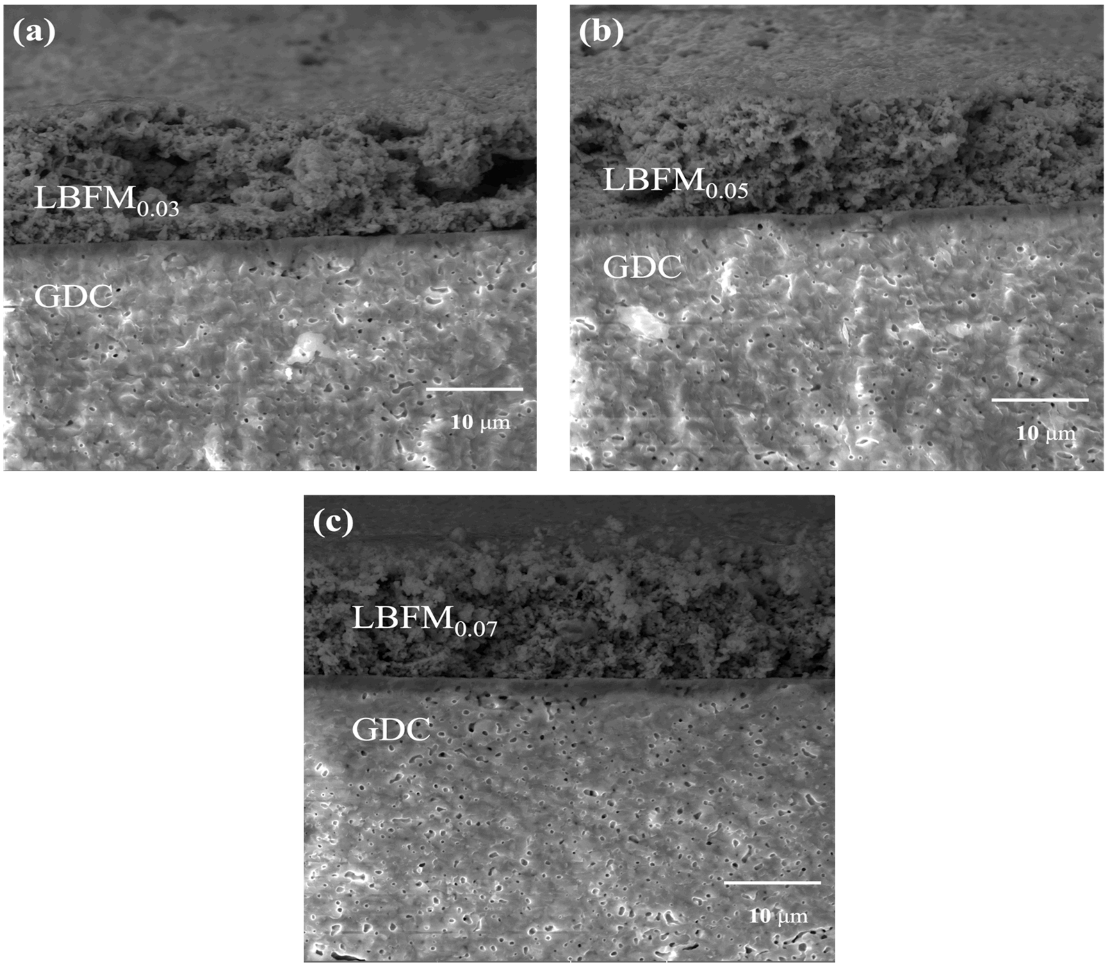

Figure S3. SEM of $LBFM_x$|CGO section（x=0.03 , 0.05 and 0.07）

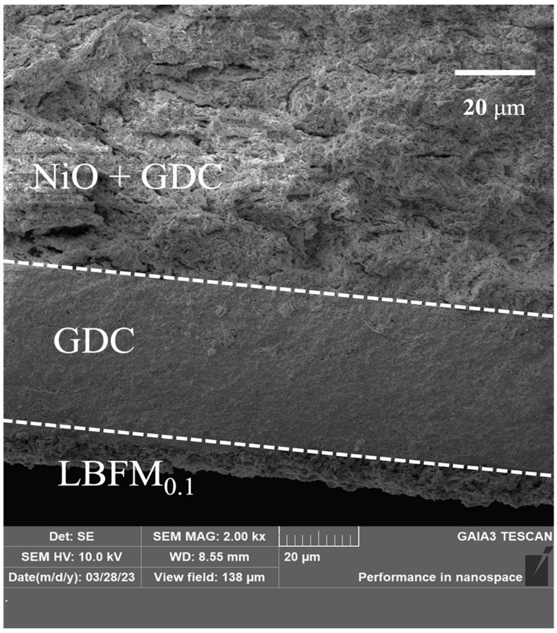

Figure S4. SEM of the single cell section $LBFM_{0.1}$|CGO|NiO+CGO

Table S1 Rietveld refinement results of $LBFM_x$

Sample		LBF	$LBFM_{0.03}$	$LBFM_{0.5}$	$LBFM_{0.07}$	$LBFM_{0.1}$
Space group		P4/mmm	P4/mmm	P4/mmm	P4/mmm	P4/mmm
	a=b(Å)	3.94095	3.945538	3.946569	3.947602	3.950509
Lattice	c(Å)	7.87891	7.879431	7.887534	7.894513	7.899870
parameters	V($Å^3$)	122.3681	122.6612	122.8516	123.0246	123.2895
		La,0,0,0.5	La,0,0,0.5	La,0,0,0.5	La,0,0,0.5	La,0,0,0.5
		Occupancy=1	Occupancy=1	Occupancy=1	Occupancy=1	Occupancy=1
		Ba,0,0,0	Ba,0,0,0	Ba,0,0,0	Ba,0,0,0	Ba,0,0,0
		Occupancy=1	Occupancy=1	Occupancy=1	Occupancy=1	Occupancy=1
		Fe,0.5,0.5,0.25	Fe,0.5,0.5,0.25	Fe,0.5,0.5,0.25	Fe,0.5,0.5,0.25	Fe,0.5,0.5,0.25
	Atom	Occupancy=1	Occupancy=0.97	Occupancy=0.95	Occupancy=0.93	Occupancy=0.90
	position		Mo,0.5,0.5,0.25	Mo,0.5,0.5,0.25	Mo,0.5,0.5,0.25	Mo,0.5,0.5,0.25
			Occupancy=0.03	Occupancy=0.05	Occupancy=0.07	Occupancy=0.10
		O1,0.5,0.5,0.5	O1,0.5,0.5,0.5	O1,0.5,0.5,0.5	O1,0.5,0.5,0.5	O1,0.5,0.5,0.5
		Occupancy=1	Occupancy=1	Occupancy=1	Occupancy=1	Occupancy=1
		O2,0,0.5,0.25	O2,0,0.5,0.25	O2,0,0.5,0.25	O2,0,0.5,0.25	O2,0,0.5,0.25
		Occupancy=1	Occupancy=1	Occupancy=1	Occupancy=1	Occupancy=1
		O3,0.5,0.5,0	O3,0.5,0.5,0	O3,0.5,0.5,0	O3,0.5,0.5,0	O3,0.5,0.5,0
		Occupancy=1	Occupancy=1	Occupancy=1	Occupancy=1	Occupancy=1
Refinement	R_p	7.74%	6.84%	3.88%	3.69%	3.97%
Parameters	R_{wp}	12.6%	12.34%	11.51%	10.62%	11.64%
	χ^2	2.679	2.245	1.632	1.54	1.654

Table S2 Oxygen content of $LBFM_x$, oxygen vacancy, average valence of Fe^{n+}

$LBFM_x$	5+δ	Oxygen vacancy	Fe^{n+} average valence state
x=0.00	5.822	0.178	3.322
x=0.03	5.811	0.189	3.270
x=0.05	5.807	0.193	3.239
x=0.07	5.793	0.207	3.195
x=0.10	5.789	0.211	3.146

Table S3 XPS percentage values for Fe and O elements

Sample	Fe^{3+}	Fe^{4+}	O_{Lat}	O_C	O_C/O_{Lat}
x=0.00	53.9	46.1	26.85	61.21	2.2797
x=0.03	55.61	44.39	26..12	62.62	2.3973
x=0.05	57.3	42.7	25.85	63.96	2.4743
x=0.07	58.05	41.95	25.82	65.88	2.5515
x=0.10	58.75	41.25	25.15	66.42	2.6409

Table S4 Average coefficient of thermal expansion of $LBFM_x$ (K^{-1})

Sample	30~300 °C	300~750 °C	30~750 °C
$LaBaFeO_{5+\delta}$	6.45×10^{-6}	21.2×10^{-6}	13.2×10^{-6}
$LaBaFe_{1.97}Mo_{0.3}O_{5+\delta}$	6.41×10^{-6}	19.4×10^{-6}	12.1×10^{-6}
$LaBaFe_{1.95}Mo_{0.05}O_{5+\delta}$	6.09×10^{-6}	18.9×10^{-6}	11.8×10^{-6}
$LaBaFe_{1.93}Mo_{0.07}O_{5+\delta}$	6.25×10^{-6}	15.6×10^{-6}	9.73×10^{-6}
$LaBaFe_{1.9}Mo_{0.1}O_{5+\delta}$	5.89×10^{-6}	14.5×10^{-6}	9.09×10^{-6}

Table S5 Polarization resistance of $LaBaFe_{2-x}Mo_xO_{5+\delta}$ ($\Omega\cdot cm^2$)

Sample	800 °C	750 °C	700 °C	650 °C	600 °C
$LaBaFe_2O_{5+\delta}$	0.04	0.07	0.124	0.267	0.802
$LaBaFe_{1.97}Mo_{0.03}O_{5+\delta}$	0.033	0.062	0.11	0.221	0.5
$LaBaFe_{1.95}Mo_{0.05}O_{5+\delta}$	0.028	0.045	0.064	0.118	0.26
$LaBaFe_{1.93}Mo_{0.07}O_{5+\delta}$	0.022	0.037	0.059	0.11	0.23
$LaBaFe_{1.9}Mo_{0.1}O_{5+\delta}$	0.017	0.025	0.04	0.07	0.14

Quantitative analysis of oxygen nonstoichiometry in $LBFM_x$ by iodometry titration

The oxygen content of $LaBaFe_{2-x}Mo_xO_{5+\delta}$ (x=0, 0.03, 0.5, 0.07 and 0.1, LBFMx) material was measured by iodine titration, assuming that the valence of Fe in the $LBFM_x$ material is y. A sample of mass 0.1 g was weighed into an iodine measuring flask, and 2 g of KI and 20 mL of dilute HCl were added, which were mixed homogeneously and then placed in the dark place for 10 min. Then measure 150 mL of water and pour it into the iodine measuring flask, and the following chemical reaction occurs in the flask:

$$Fe^{y+}+(y-2)I^{-} \rightarrow \frac{y-2}{2}I_2+Fe^{2+} \qquad \text{Equation (S1)}$$

Then use the calibrated $Na_2S_2O_3$ solution to titrate the I_2 in the solution, near the end of the titration to the iodine measuring flask to add 5 mol·L^{-1} starch solution 3 mL, until the solution becomes light blue when the end of the titration, read the volume of $Na_2S_2O_3$ solution consumed by the titration, the above experimental steps repeated three times to calculate the average value of the results obtained:

$$I_2+2Na_2S_2O_3 \rightarrow 2NaI+Na_2S_4O_6 \qquad \text{Equation (S2)}$$

V, C and M represent the volume of $Na_2S_2O_3$ solution consumed in the experiment, the concentration of $Na_2S_2O_3$ solution and the molar mass of the sample, respectively, according to equation S3:

$$CV\ (y-2)=(2-x)\frac{m}{M} \qquad \text{Equation (S3)}$$

Calculate the average valence state of Fe ions from the measured data. Other metal ion valence is known, based on the principle of conservation of charge, the positive and negative valence in the compound is ultimately equal to zero, through the titration results can be calculated to find the y value, so that the oxygen content can be found:

$$5+\delta=\frac{5}{2}+3x+(2-x)\frac{y}{2} \qquad \text{Equation (S4)}$$

where x is the Mo doping amount.
